# Exosomal Protein Biomarkers in Arthritis: Deciphering the Inflammatory Profiles of RA and OA †

**DOI:** 10.3390/biomedicines13061283

**Published:** 2025-05-23

**Authors:** Claudia M. Brenis Gómez, Chamaida Plasencia-Rodríguez, Marta Novella-Navarro, Ana Martínez-Feito, Alejandro Balsa, Enrique Calvo-Aranda, Borja Hernández-Breijo

**Affiliations:** 1 Immuno-Rheumatology Research Group, La Paz Hospital Institute for Health Research (IdiPAZ), 28046 Madrid, Spain; 2 VIB-UGent Inflammation Research Center, 9052 Gent, Belgium; 3 Rheumatology Department, La Paz University Hospital, 28046 Madrid, Spain; 4 Immunology Unit, La Paz University Hospital, 28046 Madrid, Spain; 5 Rheumatology Department, Hospital Universitario Infanta Leonor, 28031 Madrid, Spain

**Keywords:** extracellular vesicles, arthritis, rheumatoid, osteoarthritis, proteomics

## Abstract

**Background/Objectives**: Rheumatoid arthritis (RA) and osteoarthritis (OA) are highly prevalent diseases and their pathophysiology, diagnosis and treatment continue to be challenging. The aim of this study was to characterize and differentiate the profiles of the serum extracellular vesicles (EVs) isolated from RA and OA patients. **Methods**: This study included nine patients diagnosed with RA, eight patients with OA during a flare and five healthy controls (HCs). Blood samples were collected and EVs from the serum were isolated for further performance of flow cytometry and proteomic analysis. **Results**: The extracellular vesicles from HC samples exhibited smaller sizes and were more concentrated than exosomes from RA and OA samples. Surface protein expression was analyzed by flow cytometry. The results showed an enrichment of exosomes derived from antigen-presenting cells in RA samples; this was evidenced by their expression of CD14 and HLA-DR. Proteomic analysis identified 45 differentially expressed proteins between RA and OA patients. Furthermore, Ingenuity Pathway Analysis (IPA) identified inflammatory pathways such as the IL-1β and IL-6 signaling pathways as being enhanced in RA-derived exosomes, while the MYC and ROCK2 signaling pathways were enhanced in OA-derived exosomes. **Conclusions**: Our results show serum-derived exosomes from RA and OA patients harbor different surface proteins and cargo profiles, mirroring the different pathophysiologic mechanisms underlying these diseases. These results also highlight the promising use of exosomes as disease biomarkers.

## 1. Introduction

Immune-mediated conditions contribute to disability, expensive treatments, and a low quality of life. The etiology of immune-mediated diseases (with a predominance of autoimmunity or autoinflammation) is still not completely understood; however, there is evidence that genetic and environmental factors contribute to the generation of the pro-inflammatory milieu and tolerance breakage [1].

Rheumatoid arthritis (RA), which affects 0.5–1% of the population worldwide, is characterized by a chronic autoimmune inflammatory state, with symptoms like joint swelling and the destruction of synovial joints [2]. The pathogenesis of RA relies on an inflammatory response in the synovial space that is characterized by T helper type 1 cell infiltration with the further release of different cytokines, such as IL-6, TNFα, IL-1β, IL-12, IL-17, and GM-CSF; production of autoantibodies, like rheumatoid factor (RF) and anti-citrullinated peptide antibodies (ACPA), by B cells; and cytotoxic CD8 T cell activation [2]. Tissue infiltration by macrophages, T and B cells, and dendritic cells, in addition to fibroblast and osteoclast activation, leads to progressive and irreversible bone and cartilage damage and deformation [3]. Treatments aim to achieve clinical remission for a long-term decrease in structural damage and progression and to limit functional disability [3]. The treatment strategy is based on the use of disease-modifying antirheumatic drugs (DMARDs), comprising conventional (i.e., methotrexate) and biological (anti-TNFα, anti-CD20, anti-IL-6R antibodies) DMARDs. Treatment in the early stages of the disease is necessary to achieve better long-term outcomes [4,5].

Osteoarthritis (OA), which is a primary cause of disability in geriatric population, is a low-grade inflammatory disease characterized by cartilage degradation in joints, causing friction between the articular bones, with subchondral bone and synovial involvement [6]. The pathogenesis of OA is multifactorial and includes abnormal bone remodeling, chondrocyte cell death with subsequent synovial inflammation, and extracellular matrix degradation [7]. Treatment is based non-pharmacological strategies including changes in lifestyle (maintain a healthy weight, physical activity), the use of topical or oral analgesics and intraarticular steroid or hyaluronic acid injections [8,9].

Both conditions contribute to disability, expensive treatments, and a low quality of life. Even though these pathologies have different pathophysiologic backgrounds and extensive diagnostic protocols, it continues to be challenging to differentiate them, especially in early stages of disease. When clinical manifestations are mild and non-specific, there is no conclusive evidence of radiographic changes and RA patients have negative RF and ACPA [10]. Research has shown that patients with RA benefit the most when treatment is started in the early stages of the disease, the so-called “window of opportunity” [11], which highlights the need for better diagnostic tools and biomarkers to identify the disease at early stages.

In relation to this, intercellular communication is a key feature of physiological and pathological mechanisms, where extracellular vesicles (EVs) play an interesting role in autocrine and paracrine communication, performing a wide range of modulatory actions. Three types of EVs have been identified: apoptotic bodies, microvesicles (MVs, also called ectosomes) and exosomes. They are formally classified by their size; however, there are several differences between them in relation to their pathway of production and release, their cargo and surface markers. EVs are involved in several cellular mechanisms and physiological processes, such as neuronal communication, antigen presentation, tissue remodeling, organogenesis and the immune response [12]. Exosomes carry a complex and heterogeneous cargo that is related to the characteristics of their parent cell and the microenvironment, making exosome content specific to the origin cell type. Different molecules have been found on their surfaces and inside them, like proteins, lipids and microRNA, and have functions in wide range of processes, such as adhesion, the cell cycle and gene and immune regulation [13,14]. Previous research has pointed to their use as biomarkers in early-stage cancer and neurodegenerative diseases [15,16]. Their easy access through blood sampling, in addition to their specific cargo, makes them suitable as noninvasive biomarkers of disease.

In this exploratory study, we aimed to identify the cellular process occurring in inflamed tissues by isolating circulating EVs from patients with RA and OA. Using flow cytometry and proteomic analysis we identified differences in the surface characteristics and content between RA- and OA-derived EVs.

## 2. Materials and Methods

### 2.1. Subjects

A bicenter cohort study was conducted between September 2020 and June 2021. Nine patients with RA and eight patients with OA were recruited. Patients that fulfilled the diagnostic criteria of the American College of Rheumatology/European League Against Rheumatism were included [3,6]. Individuals without any chronic or acute illness were included as healthy volunteers. All patients and healthy volunteers signed an informed written consent prior to participation in the study. The study was approved by La Paz and Gregorio Marañón University Hospital Ethics Committees (PI-2800).

### 2.2. Blood Samples

Peripheral blood samples were collected when patients arrived at the emergency room or external consultation for a disease flare at La Paz University Hospital or Infanta Leonor University Hospital in Madrid. Demographic and analytical variables were systematically collected in a database by means of an electronic CRF at the Biologic Unit of La Paz University Hospital and Infanta Leonor University Hospital. Laboratory tests for C-reactive protein (CRP), rheumatoid factor (RF) and anti-citrullinated peptide antibody (ACPA) were performed.

Serum samples were obtained by centrifugation (2000× *g* during 20 min) and stored at −80 °C in our sample collection (no. C.0004887, registered at National Biobank Register of Health Institute Salud Carlos III). Serum samples from healthy volunteers were included as controls. Exosome isolation and further analysis were all performed at La Paz University Hospital.

### 2.3. Exosome Isolation

Serum samples were centrifuged at 1500× *g* for 10 min at 4 °C. The resulting supernatant was centrifuged at 12,000× *g* for 20 min at 4 °C to remove apoptotic bodies and microvesicles. For the exosome isolation, size exclusion chromatography (SEC) [17] was performed using EVs SEC 70 nm columns (Immunostep S.L, Salamanca, Spain) following the manufacturer’s instructions. Briefly, columns were rinsed with 15 mL of 0.2 µm filtered and degassed PBS prior to every experiment. An amount of 500 µL of the sample was added, then SEC was run in PBS. The first 3 mL obtained, which corresponded to the column void volume, was discarded, obtaining the next 1.5 mL. Fresh isolated exosome samples were used for further experiments.

### 2.4. Nanotracking Analysis

Nanosight LM10 (Malvern Panalytical Ltd., Salisbury, UK) was used, following the manufacturer’s recommendations. The Nanosight was washed first with 10 mL distilled water to remove all possible contaminant particles. An amount of 200 µL of the isolated exosomes were careful injected into the system. Three 60 s video captures were taken for each sample. All captures were taken with a screen gain of 10 and a detection threshold of 4. The minimum exosome concentration required for a good capture was considered as >10 particles/frame.

### 2.5. Flow Cytometry

For flow cytometry characterization, EVs were captured with CD63-coated beads (ExoStepTM Exosome capture and detection kit, Immunostep S.L, Salamanca, Spain). The experiment was conducted following the manufacturer’s instructions. Briefly, 100 μL of isolated exosomes (patients or controls) was mixed with 50 μL capture beads and incubated overnight at room temperature in the dark. To remove the unbound beads, samples were washed with 1 mL of Assay Buffer (PBS + 1% BSA) and centrifuged at 2500× *g* for 5 min. The supernatant was removed and discarded. Next, bead-bound exosomes were labeled with 5 μL of biotinylated anti-CD9, anti-CD14, anti-CD19, anti-CD3 or anti-HLA-DR antibodies (each antibody in different tube) and then incubated (60 min at 4 °C in the dark). The samples were washed again with Assay Buffer and centrifuged at 2500× *g* for 5 min. Supernatant was then removed. Samples were mixed with 5 μL of Strp-PE for a final incubation (30 min at 4 °C protected from light). Samples were centrifuged 5 min at 2500× *g* after being previously washed with Assay Buffer. After the removal of the supernatant, samples were resuspended in 350 μL of Assay Buffer. Additional tubes without exosomes and without anti-CD9 antibody were prepared as negative controls. A tube with an IgG isotype was prepared to determinate if there was unspecific antibody binding. The samples were analyzed in the Navios EX Flow Cytometer (Beckman Coulter, Inc., Brea, CA, USA). Flow cytometry analysis was performed using Kaluza Analysis Software V 2.1 (Beckman Coulter, Inc., USA). The Relative Fluorescence Intensity (RFI) index was calculated using MFI positive/MFI background (negative control).

### 2.6. Exosome Concentration and Lysis

The exosome samples were concentrated using a Pall Nanosep centrifugal device with a 10 kDa Omega membrane (Pall Corporation, Port Washington, NY, USA). First, the centrifugal devices needed to be passivated with 500 µL of SDS (5%) for 1 h and then centrifuged at 4000× *g* for 5 min before their utilization. Samples were pipetted into the upper reservoir of the device, then the samples were centrifuged at 14,000× *g* at 4 °C until 70 µL of concentrated exosomes was obtained in the upper reservoir. Samples were lysed with 30 µL of 20% SDS plus a broad-spectrum cysteine and serine proteases inhibitor cocktail that was EDTA-free (Roche, Basel, Switzerland) and then incubated 10 min at 95 °C. Samples were mixed by pipetting and incubated on ice for 5 min. Sample were sonicated for 10 s at room temperature and incubated for 1 min on ice 4 times. Samples were centrifuged 12,000× *g* for 10 min at 4 °C and then stored at −20 °C.

### 2.7. Mass Spectrometry

Mass spectrometry was only performed on RA and OA samples. The procedure was conducted as previously described [18]. The mass spectrometry analysis was performed with LC-MS/MS Exploris 240 with an Orbitrap analyzer (Thermofisher, Waltham, MA, USA) mass spectrometer in collaboration with proteomic laboratory from Biotechnology National Center, Madrid. First, proteins derived from the exosome lysates were precipitated with MeOH/chloroform and quantified by Pierce 660 nm Protein Assay (Thermofisher, USA). Pierce 660 nm is based on a red dye–metal complex that interacts with basic residues on proteins, changing its color to green to further absorbance measurements at 660 nm. The experiment was performed following manufacturer’s instructions. Briefly, 10 µL of sample was mixed with 150 µL of protein assay reagent and incubated for 5 min at room temperature; the absorbance was measured at 660 nm and compared to a standard curve. Next, exosome lysates were reduced with tris-(2-carboxyethyl) phosphine (TCEP) and alkylated with 200 mM methylmethane-thiosulfonate (MMTS). Therefore, tryptic digestion was performed using S-Trap columns (ProtiFi, Fairport, NY, USA). An amount of 1 ug of tryptic was injected into the LC-MS/MS system. The chromatographic conditions were a flow rate of 250 nL/min, with an oven temperature of 50 °C. The mass spectrometer identifies ions in the *m*/*z* range of 300–1200. The 25 most intense ones are selected to obtain their MS/MS fragmentation spectra and identify those peptides. Mass spectrometry was performed with 10 precursor ions with an accumulation time of 250 ms in MS1 and 100 ms per precursor ion in MS2 as acquisition parameters. Peptides are identified by their mass spectra comparison with a known human protein database. Mascot Server v. 2.3 (Matrix Science, London, UK) was used as a search engine, which allows us to compare the fragmentation spectra MS/MS of the sample with the UniProt-SwissProt database. Results were filtered at the peptide level to a False Discovery Rate <1%. Functional analysis was performed with Ingenuity Pathway Analysis (IPA) software V 21.0 (Qiagen, Venlo, Netherlands).

### 2.8. Statistical Analysis

Descriptive analyses were performed for the demographic and clinical variables. Differences in qualitative variables were assessed using the Fisher’s exact test. Comparisons of continuous data were conducted using the unpaired *t*-test or Mann–Whitney U test, depending on data distribution. For multiple comparisons, one-way ANOVA (post hoc Bonferroni test) or the Kruskal–Wallis (plus post hoc Dunn’s test) test were used. A *p*-value < 0.05 was considered statistically significant. Data were analyzed using GraphPad Prism 6 software (GraphPad Software V 6, USA) and the SPSS statistical software package V 28.0.1 (IBM, Armonk, NY, USA).

## 3. Results

The healthy control group comprised three (60%) females and two (40%) males with a median age of 26 (31–24). Patient characteristics are summarized in Table 1.

The results show patients in the RA group were mostly females (89% vs. 25%, *p*-value < 0.01), in contrast with OA group. There was no significant difference in age, body mass index (BMI), duration of the disease, C-reactive peptide titers (CRP) and smoking habit between both groups. Eight out of nine RA patients were positive for both rheumatoid factor and anti-citrullinated peptide antibody.

**Figure 1:**
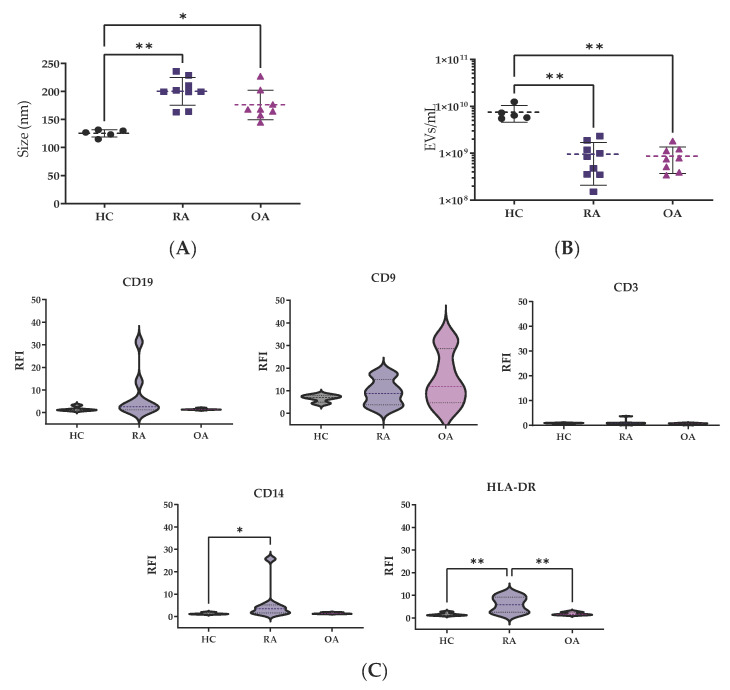
EV characterization by nanotracking analysis. Differences in (**A**) mode size (nm) and (**B**) EV concentration were analyzed among the groups. Values are shown as mean ± SD. (**C**) RFI of surface markers. The Kruskal–Wallis and post hoc Dunn’s tests were performed to assess statistical significance. * *p* < 0.05; ** *p* < 0.01. RFI: relative fluorescence index.

After EV isolation through SEC, EV fractions were analyzed by nanotracking analysis for concentration and size determination. The size measurements from HCs were quite homogeneous, showing a median size of 127.2 nm (119.4–131.1), while bigger EV sizes were observed in patients with RA 199.9 nm (181.9–219.9, *p* < 0.01) and OA 168.1 nm (159.5–196.1, *p* < 0.05) in comparison to HCs (Figure 1A).

However, patients showed lower EVs concentration, OA 0.7 × 10^9^ (0.4 × 10^9^–1.1 × 10^9^ particles/mL) and RA 0.8 × 10^9^ (0.4 × 10^9^–1.2 × 10^9^ particles/mL), in comparison to HCs 6.5 × 10^9^ (0.3 × 10^9^–1.5 × 10^9^ particles/mL) (*p* < 0.05, both comparisons) (Figure 1B).

For surface characterization of EVs, CD9 was used as a control marker (Figure 1C). The relative fluorescence index (RFI) from CD14 for monocyte-derived EVs was higher in RA samples in comparison to HC and OA samples (*p* < 0.05). There were no significant differences between the CD3 or CD19 markers for T and B cells among the groups. The HLA-DR levels for EVs derived from antigen-presenting cells, such as dendritic cells (DCs), were clearly higher in RA patients in comparison to HC and OA (*p* < 0.01) EV samples.

To ensure the purity of the EVs, the proteome found in our samples was paired with a list of the 34 most commonly expected proteins (VesiclePedia database). We found 27 out of these 34 proteins; therefore, the analyzed sample had enough purity. Table 2 [19] shows the 45 differentially expressed proteins between the RA and OA samples. The exosomes from OA patients were high in proteins such as Annexin A2 (ANXA2), filaggrin (FLG) and fatty acid-binding protein (FABP5), while RA-patient-derived exosomes showed a higher cargo of alpha-2-macroglobulin (A2M), apolipoprotein B (APOB) and fibronectin (FN1) proteins.

**Figure 2:**
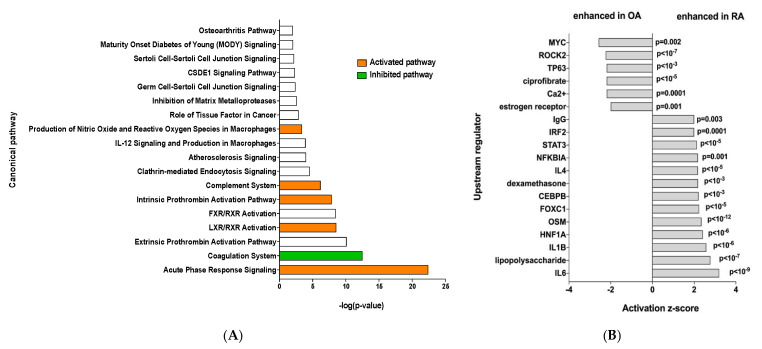
(**A**) Ingenuity Pathway Analysis, orange bar = positive z-score, white bar = z-score 0, green bar = negative z-score and (**B**) IPA upstream regulator analysis of the 45 differentially expressed proteins.

Ingenuity Pathway Analysis was performed and showed that RA EV proteins have an enrichment for the “acute phase response signaling”, “Liver X receptor/retinoid X receptor (LXR/RXR) activation” and “complement system” pathways, showing a clear inflammatory signature (Figure 2A). To identify the specific signaling pathways involved, an IPA upstream regulator analysis was performed. This showed enrichment of more inflammatory pathways such as the IL6 and IL1b signaling pathways, among others, while OA samples showed enrichment of the MYC and ROCK2 pathways (Figure 2B).

## 4. Discussion

In this study we investigated the size, concentration, surface, and proteomic profile of EVs from serum samples of patients with RA and OA as well as HCs. The chosen EV isolation method was size exclusion chromatography, which allows us to obtain a pure and rich sample of exosomes in a size range of 70 to 1000 nm without modifying their shape and function. International guides (ISEV) propose the usage of two different methods to verify correct EV isolation [20]; with that in mind, we characterized our samples with nanotracking analysis (NTA), flow cytometry and mass spectrometry.

Previous studies have reported heterogeneity in EV sizes and concentrations measured using NTA, reporting exosomes in serum and synovial fluid with bigger sizes in patients with RA and other autoimmune diseases. Similar to previous authors, we identified bigger EV sizes in patients in contrast to HCs, who have smaller and homogeneous vesicles. Some authors [21] have suggested that smaller exosomes are released by platelets while bigger ones are release by leukocyte. Supporting our results, patients with more inflammatory and systemic diseases, like RA, had bigger EV diameters. Patients had lower EV concentrations than HCs, this result has been reported previously and attributed to the formation of immune complexes, which are well-known participants in seropositive RA pathology [13,14]. In addition, the difference in concentration levels could be attributed to the disparity of age between the HCs and patients. Previous research has proposed an age-related decrease in the concentration of EVs that is attributed to increased internalization by leucocytes [22].

Although SEC is a very specific isolation method, there is no method available that allows a 100% pure isolation [20]. Our results confirm the accuracy of the isolation procedure used, reflected by the binding to CD63 beads as well as CD9 [23,24]. Leukocyte markers showed clear differences among patients with RA or OA and HCs. Monocytes and macrophages are known to be key players in RA pathogenesis, releasing high amounts of proinflammatory cytokines like IL-6 and IL-1b, and this is clearly reflected in our results [25].

Previous articles show a huge T cell infiltration in the synovial fluid of RA patients, which plays a key role in RA pathogenesis; however, our results did not seem to mirror the joint inflammatory environment in relation to T lymphocytes. Further research is needed in order to reach definitive conclusions. We observed a clear difference between RA patients and the other two groups, showing RA patients had higher levels of DC-derived EVs (HLA-DR positive). This finding is supported by previous studies involving the serum and synovial fluid of patients with RA, which also found higher levels of HLA-DR+ EVs [26]. Although HLA-DR+ EVs are mainly released by DCs, they could also be released by monocytes or B cells, evidencing the clear autoinflammatory environment in RA.

Proteomic analysis confirmed that a good isolation method was used. The samples showed an enrichment in exosome-related proteins like tetraspanin CD63, endosomal origin proteins like programmed cell death 6-interacting protein, a protein that allows the interaction between ESCRT-0 and ESCRT-II; Rab proteins, which are conducive to exosome release; proteins that allow exosome uptake like clathrin; and common cargo such as heat shock proteins, 14-3-3 proteins and annexins [23].

Some of the differentially expressed proteins found in RA patients have been previously reported in the literature. APOA1 has been found in the synovial tissue of RA patients, with a correlation between protein level and activity of the disease. APOB has been shown to bind the enolase-1 present on monocytes and stimulate the production of IL-1b, IL-6 and TFNa, amplifying synovial inflammation [27,28]. Tenascin-C is an extracellular matrix glycoprotein that is correlated with tissue inflammation and repair. High serum levels of this protein have been found in patients with RA, as well as those with other autoimmune diseases like systemic lupus erythematosus, idiopathic inflammatory myositis, psoriatic arthritis and ankylosing spondylitis [29,30]. Within the differentially expressed proteins, we also found that fibronectin 1 was present in RA samples; this result correlates with previous research that shows citrullinated fibronectin in the inflamed synovial tissue of RA patients [31]. IPA analysis further confirmed that RA samples had an enrichment in inflammatory signaling pathways.

Our results show that the EVs obtained from routine blood tests carry surface markers that may be evidence of their cell of origin, thus correlating with the pathophysiology of the disease. The EV cargo found by proteomic analysis showed a variety of proteins previously described as biomarkers in the inflamed tissue of RA patients. Taken together, these results point to a promising use of EVs as biomarkers in inflammatory diseases.

This study demonstrates structural and proteomic differences between immune-mediated diseases and healthy controls; however, there are some limitations that should be mentioned that are mainly based on the preliminary stage of the project. The limitations of the study included the heterogeneity of age, duration of disease and treatment received, so we cannot completely rule out that these factors could have an effect on the results obtained. Second, it is important to consider the low number of patients (samples) included in the study; further research should be conducted with higher number of participants. Third, we characterized exosomes from peripheral blood and not synovial fluid. While it has been described that the exosomes obtained from serum may reflect the pathology happening in other tissues, a proper study including both serum and synovial fluid should be conducted to sustain that hypothesis.

## 5. Conclusions

Our results show the different profiles of serum-purified EVs from RA and OA patients. OA and RA patients had larger and less concentrated serum EVs than their healthy counterparts. Flow cytometry analysis revealed a higher abundance of EVs derived from immune cells, correlating with the pathophysiology of these rheumatic diseases. Proteomic analysis identified biomarkers that are supported by the previous literature. Further research in bigger cohorts needs to be conducted to explore the utility of these biomarkers in early RA diagnosis. These results suggest EVs as a promising source of disease-protein biomarkers that could lead to early detection and treatment of patients with rheumatic diseases.

## Figures and Tables

**Table 1:** Patient characteristics.

Patient Characteristics	Rheumatoid Arthritis (n = 9)	Osteoarthritis (n = 8)	*p*-Value
Female, n (%)	8 (89)	2 (25)	<0.01
Age (yr)	60 ± 11	68 ± 11	0.1
BMI (kg/m^2^)	30.0 ± 5.3	31.0 ± 2.0	0.9
Disease duration (yr)	5 (1–13)	0 (0–17)	0.4
CRP (mg/dL)	5.0 (4.1–92.3)	6.6 (4.4–43.6)	0.8
Smokers, n (%)	4 (44)	5 (62)	0.6
Seropositivity, n (%) *	8 (89)		
RF titer (AU/mL)	127 (34–465)		
ACPA titer (AU/mL)	300 (191–748)		

Values are shown as mean ± SD, median (interquartile range) or absolute number (percentage) for all patients (n = 17). * The values of seropositivity, ACPA and RF correspond to RA patients. Kruskal–Wallis test was performed for scalar variables and Fisher’s exact test for categorical variables. A *p*-value < 0.05 was considered as statistically significant. BMI, body mass index; CRP, C-reactive protein; RF, rheumatoid factor: ACPA, anti-citrullinated peptide antibody.

**Table 2:** The 45 differentially expressed proteins between RA- and OA-derived EVs.

Increased in OA (Gene Symbol)	Increased in RA (Gene Symbol)	Log_2_ (RA/OA)	q-Value
ANXA2		−7.6	<0.05
FLG		−6.5	<0.05
FABP5		−6.2	<0.1
SPRR1B		−5.0	<0.1
ASPRV1		−4.7	<0.1
SFN		−4.7	<0.05
JUP		−4.2	<0.1
DSG1		−4.0	<0.1
KRT5		−3.6	<0.05
IGHV1-8		−3.5	<0.1
S100A7		−3.4	<0.1
IGKV1D-13		−3.3	<0.1
TUBA1A		−3.1	<0.1
DSP		−2.7	<0.05
KRT1		−1.3	<0.01
	A2M	0.4	<0.1
	APOB	0.4	<0.01
	FN1	0.6	<0.05
	APOA1	0.8	<0.1
	PROS1	1.0	<0.05
	C4BPA	1.1	<0.01
	CLU	1.2	<0.1
	F2	1.4	<0.01
	C1S	1.5	<0.1
	FCGBP	1.5	<0.01
	TNC	1.6	<0.1
	SERPINC1	1.6	<0.01
	C1R	1.7	<0.01
	VTN	1.8	<0.05
	LRP1	1.9	<0.05
	SERPINA1	1.9	<0.1
	HRG	2.0	<0.05
	SERPINA3	2.4	<0.1
	MBL2	3.0	<0.1
	PCSK9	3.4	<0.05
	FGB	3.4	<0.01
	FGG	3.6	<0.05
	SAA2-SAA4	3.7	<0.05
	IGLV3-9	3.7	<0.1
	PZP	3.8	<0.05
	IGHV4-34	3.9	<0.1
	SAA2	4.6	<0.1
	SAA1	4.7	<0.01
	IGKV6-21	4.7	<0.1
	LRP10	5.4	<0.1

## Data Availability

The original contributions presented in this study are included in the article. Further inquiries can be directed to the corresponding authors.
